# Generalized Peritonitis Secondary to Spontaneous Perforation of Pyometra in a 63-Year-Old Patient

**DOI:** 10.1155/2013/929407

**Published:** 2013-08-29

**Authors:** Ahmed Abu-Zaid, Osama AlOmar, Ahmed Nazer, Ayman Azzam, Zainab Abudan, Ismail Al-Badawi

**Affiliations:** ^1^College of Medicine, Alfaisal University, P.O. Box 50927, Riyadh 11533, Saudi Arabia; ^2^Department of Obstetrics and Gynecology, King Faisal Specialist Hospital and Research Center (KFSH&RC), P.O. Box 3354, Riyadh 11211, Saudi Arabia; ^3^Department of General Surgery, Faculty of Medicine, Alexandria University, Alexandria 21526, Egypt

## Abstract

Spontaneous perforation of pyometra resulting in generalized diffuse peritonitis is extremely uncommon. Herein, we report the case of a 63-year-old woman who presented to emergency department with a 2-day history of severe diffuse abdominal pain, high-grade fever, nausea, and vomiting. Acute abdomen series was done, and upright plain chest radiograph showed free air under diaphragm. A noncontrast-enhanced computed tomography scan showed a significantly distended fluid-filled uterus measuring 10 × 7.8 × 10 cm, in addition to a single focus of perforation involving the uterine fundus and associated with presence of free air within the nondependant area. No evidence of ascites or pelvi-abdominal lymphadenopathy was identified. A preoperative diagnosis of generalized peritonitis secondary to spontaneous perforation of uterus was established. Subsequently, patient underwent urgent exploratory laparotomy which revealed pus-filled uterus with perforated fundus. Diagnosis of generalized peritonitis secondary to spontaneous perforation of pyometra was established. Consequently, patient underwent total abdominal hysterectomy with bilateral salpingo-oophorectomy, as well as thorough drainage and irrigation of pelvi-abdominal cavity. Postoperatively, patient was admitted to intensive care unit. Histopathological examination of uterus was negative for malignancy, and surgical culture grew *Streptococcus constellatus*. Patient had an uneventful recovery. Moreover, a brief literature review on pyometra is presented.

## 1. Introduction

Pyometra is defined as buildup of pus (purulent material) in the uterine cavity [1]. Its reported incidence in gynecologic clinics ranges from 0.5% in young premenopausal women and increases to 13.5% in elderly postmenopausal women [2]. Spontaneous perforation of pyometra resulting in generalized diffuse peritonitis is extremely uncommon, and its reported gynecologic incidence varies from 0.01 to 0.05% [3]. Less than 50 case reports of spontaneous perforation of pyometra have been documented in the English literature so far. Herein, we report the case of a 63-year-old woman who presented to emergency department with a 2-day history of severe diffuse abdominal pain, high-grade fever, nausea, and vomiting. Patient underwent urgent exploratory laparotomy which confirmed diagnosis of generalized peritonitis secondary to spontaneous perforation of pyometra. In addition, a brief literature review on pyometra is presented.

## 2. Case Report

A 63-year-old woman presented to emergency department with a 2-day history of severe diffuse abdominal pain, high-grade fever, nausea, and vomiting. Patient was sexually active and denied any recent history of abnormal bleeding, spotting episodes, or vaginal discharges. Past medical history and surgical history were unremarkable. Systemic review was remarkable for decreased appetite and fatigue.

On physical examination, she looked ill and in pain. Her vitals were as follows: temperature of 38.7°C, blood pressure of 104/71 mmHg, heart rate (pulse) of 116 beats/minute, respiratory rate of 23 breaths/minute, and oxygen saturation of 97% in room air. Abdomen was slightly distended with positive guarding and positive rebound tenderness. Per vaginal examination showed no cervical/vaginal anomalies, vaginal discharge, or detectable pelvic mass. Initial laboratory results showed a white blood count (WBC) of 27 × 10^9^/L (high neutrophilia), lactic acid of 2.1 mmol/L, and normal hepatic, coagulation, bone, renal, and electrolyte profiles. 

**Figure 1:**
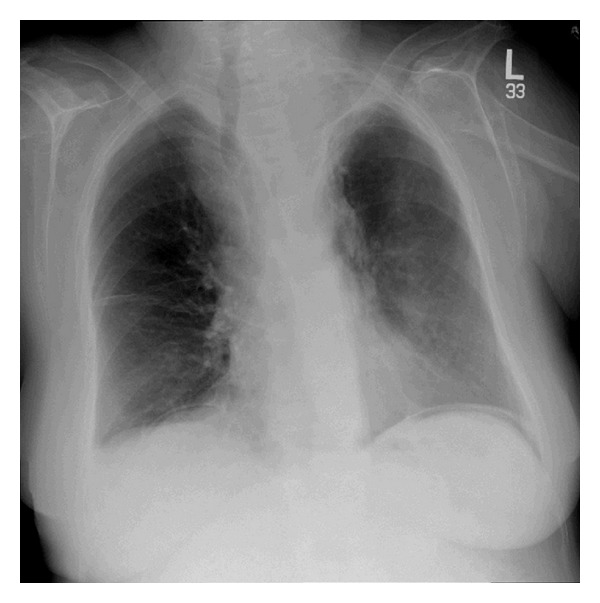
Upright chest plain radiograph (X-ray) showing free air (gas) under diaphragm.

**Figure 2:**
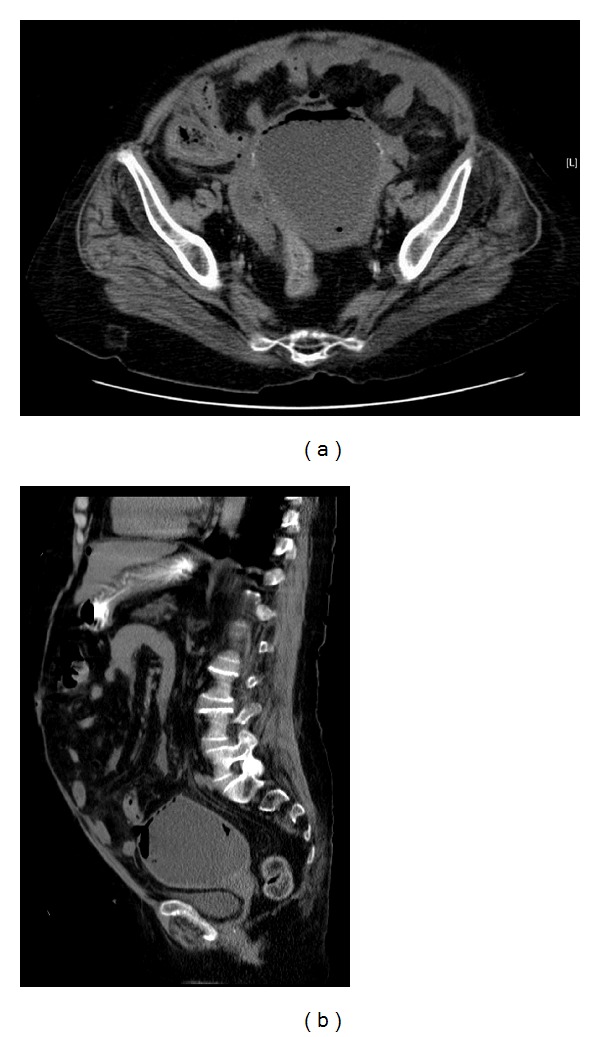
Transverse (a) and sagittal (b) views of noncontrasted-enhanced computed tomography (CT) scan showing a significantly distended fluid-filled uterus measuring 10 × 7.8 × 10 cm, in addition to a single focus of perforation involving the uterine fundus and associated with presence of free air within the nondependant area. No evidence of ascites or pelvi-abdominal lymphadenopathy was identified.

Acute abdomen series was done, and upright plain chest radiograph (X-ray) showed free air (gas) under diaphragm (Figure 1). A noncontrast-enhanced computed tomography (CT) scan showed a significantly distended fluid-filled uterus measuring 10 × 7.8 × 10 cm, in addition to a single focus of perforation involving the uterine fundus and associated with presence of free air within the nondependant area. No evidence of ascites or pelvi-abdominal lymphadenopathy was identified (Figures 2(a) and 2(b)). A preoperative diagnosis of generalized peritonitis secondary to spontaneous perforation of uterus was established. Subsequently, patient underwent urgent exploratory laparotomy which revealed pus-filled uterus with perforated fundus. Diagnosis of generalized peritonitis secondary to spontaneous perforation of pyometra was established. Consequently, the patient underwent total abdominal hysterectomy with bilateral salpingo-oophorectomy, as well as thorough drainage and irrigation of pelvi-abdominal cavity. Postoperatively, patient was admitted to intensive care unit and administered broad-spectrum antibiotics. Histopathological examination of uterus was negative for malignancy, and surgical culture grew *Streptococcus constellatus* organism which was sensitive to penicillin and ampicillin. 

Patient had an uneventful recovery afterwards and was discharged on the 15th postoperative day without complications.

## 3. Discussion

Pyometra is defined as buildup of pus (purulent material) in the uterine cavity [1]. It is an exceedingly unusual gynecologic condition that happens mostly in postmenopausal age group and hardly ever in premenopausal age group [4]. Its reported incidence in gynecological clinics ranges from 0.5% in young premenopausal women and increases to 13.5% in elderly postmenopausal women [2]. 

Pyometra primarily results from obstruction of cervical canal which in turn interferes with its natural drainage and increases susceptibility to infections leading to accumulation of pus and bloody material [1]. Possible etiologies are many and include benign or malignant gynecologic neoplasms, cervical stenosis, atrophic cervicitis, senile cervicitis, gynecology related postoperative complications, postradiation therapy, congenital malformations (anomalies), uterine infections, age-related endometritis, puerperal endometritis with retention of lochia, use of intrauterine device (IUD), and others [1, 5, 6]. The incidence of concomitant gynecologic malignancy as an underlying etiology for pyometra is considerably high [4], particularly in elderly postmenopausal women [7]. 

Potential predisposing risk factors for pyometra are numerous and include coexistence of comorbid chronic illnesses (e.g., diabetes, osteoarthritis), restricted mobility, bowel incontinence, malnutrition, poor hygiene, compromised immunity, excessive sexual activity, age-related genital tract atrophy, and uterine circulatory insufficiency—all of which lead to increased vulnerability to uterine infections and subsequent pus formation [1, 4]. Long-term use of IUD is also a significant well-recognized risk factor for pyometra [8]. Generally, the most common microorganisms causing pyometra are staphylococci, streptococci, and coliforms [1].

Determining the definitive diagnosis of pyometra preoperatively is largely difficult [9]. This is because nearly more than 50% of nonperforated pyometra patients are asymptomatic [10]. The most frequently presenting symptoms are purulent vaginal discharge, postmenopausal vaginal bleeding/spotting, lower abdominal/suprapubic spastic (cramping) pain, and uterine distension [1, 6]. Nausea, vomiting, and fever can also happen [1, 6, 8]. These symptoms, however, are nonspecific [9, 11], resulting in postponed or even missed diagnosis and subsequently increased probability to perforation of pyometra—if not detected at early stages [9, 11].

Spontaneous perforation of pyometra is exceedingly rare [3]. Using PubMed search, less than 50 case reports have been documented in the English literature so far. Spontaneous perforation of pyometra resulting in generalized diffuse peritonitis is extremely uncommon, and its reported gynecologic incidence varies from 0.01 to 0.05% [3]. Once pyometra is ruptured, morbidity and mortality increase, clinical symptoms become much more severe in intensity, and acute abdomen occurs which most often requires prompt emergency surgical intervention (i.e., exploratory laparotomy) [12]. Septic shock is a one possible life-threatening complication [13]. The most frequent preoperative diagnoses are generalized peritonitis, pneumoperitoneum, and gastrointestinal tract perforation [1].

Definitive preoperative diagnosis of spontaneous perforation of pyometra is very infrequent [12] and requires high index of clinical suspicion in addition to aided imaging studies. Imaging studies, particularly abdominal plain radiograph, sonography, and computed tomography (CT) scan, play significant roles in establishing diagnosis of spontaneous perforation of pyometra [11, 14]. Abdominal plain radiograph (X-ray) may demonstrate pneumoperitoneum and free air (gas) under diaphragm. Pelvi-abdominal sonography may display fluid accumulation in the uterine and/or peritoneal cavities. Contrast-enhanced abdominal computed tomography (CT) scan may illustrate pneumoperitoneum, air-fluid levels in uterine cavity, fluid-filled uterus, uterine wall perforation, and intraperitoneal fluid collection communicating with the intrauterine fluid through the uterine wall perforation (defect). Uterine fundus is the most frequently implicated site in perforation of pyometra during imaging and laparotomy [1].

Management of pyometra depends on the clinical setting (emergency or nonemergency) and status of pyometra (ruptured or nonruptured). Urgent and ruptured pyometra cases should be managed by total abdominal hysterectomy with bilateral salpingo-oophorectomy, thorough drainage and irrigation of pelvi-abdominal cavity, postoperative intensive care support, and administration of broad-spectrum antibiotics [11, 14, 15]. Conversely, nonurgent and most importantly nonruptured pyometra cases can be managed successfully by transcervical drainage and irrigation of uterus and subsequent administration of broad-spectrum antibiotics and supportive treatment [16].

## 4. Conclusion

Pyometra, an extremely rare gynecologic condition, is defined as build up of pus (purulent material) in uterine cavity. Although exceedingly uncommon, spontaneous perforation of pyometra should be considered in the differential diagnosis of any elderly postmenopausal woman presenting with acute abdomen and signs of generalized peritonitis. Only around 50 case reports of spontaneous perforation of pyometra have been documented in the English literature so far. Urgent exploratory laparotomy is fundamental in confirming diagnosis and successfully managing patient. Abdominal hysterectomy with bilateral salpingo-oophorectomy, thorough drainage and irrigation of pelvi-abdominal cavity, postoperative intensive care support, and administration of broad-spectrum antibiotics is the mainstay of management in emergency settings. An underlying gynecologic malignancy is considerably high in pyometra and must be ruled out.

## References

[1] Yildizhan B, Uyar E, Sişmanoğlu A, Güllüoğlu G, Kavak ZN (2006). Spontaneous perforation of pyometra. *Infectious diseases in obstetrics and gynecology*.

[2] Sawabe M, Takubo K, Esaki Y, Hatano N, Noro T, Nokubi M (1995). Spontaneous uterine perforation as a serious complication of pyometra in elderly females. *Australian and New Zealand Journal of Obstetrics and Gynaecology*.

[3] Gita R, Jain K, Vaid NB (1995). Spontaneous rupture of pyometra. *International Journal of Gynecology and Obstetrics*.

[4] Chan LY, Lau TK, Wong SF, Yuen PM (2001). Pyometra: what is its clinical significance?. *Journal of Reproductive Medicine for the Obstetrician and Gynecologist*.

[5] Tai C-C, Lien W-C, Wang H-P, Liu K-L (2007). Early diagnosis of gas-forming pyometra in an aged patient can prevent mortality. *The American Journal of Emergency Medicine*.

[6] Imachi M, Tanaka S, Ishikawa S, Matsuo K (1993). Spontaneous perforation of pyometra presenting as generalized peritonitis in a patient with cervical cancer. *Gynecologic Oncology*.

[7] Ikematsu Y, Kitajima T, Kamohara Y (1996). Spontaneous perforated pyometra presenting as pneumoperitoneum. *Gynecologic and Obstetric Investigation*.

[8] Li C-H, Chang W-C (2008). Spontaneous perforated Pyometra with an intrauterine device in menopause: a case report. *Japanese Journal of Infectious Diseases*.

[9] Ou Y-C, Lan K-C, Lin H, Tsai C-C, Changchien C-C (2010). Clinical characteristics of perforated pyometra and impending perforation: specific issues in gynecological emergency. *Journal of Obstetrics and Gynaecology Research*.

[10] Hansen PT, Lindholt J (1985). Spontaneously perforated pyometra: a differential diagnosis in acute abdomen. *Annales Chirurgiae et Gynaecologiae*.

[11] Agarwal R, Suneja A, Sharma A, Vaid NB (2011). An unusual etiology of spontaneous pyometra perforation: a case report. *Journal of Reproduction and Infertility*.

[12] Geranpayeh L, Fadaei-Araghi M, Shakiba B (2006). Spontaneous uterine perforation due to pyometra presenting as acute abdomen. *Infectious Diseases in Obstetrics and Gynecology*.

[13] Bangal VB, Giri PA, Singh RK (2012). A rare case of peritonitis following spontaneous rupture of Pyometra. *Journal of Basic and Clinical Reproductive Sciences*.

[14] Khan A, Prasad J (2012). Perforated pyometra presenting as a pelvic abscess: a case report. *International Journal of Case Reports and Images*.

[15] Nakao A, Mimura H, Fujisawa K (2000). Generalized peritonitis due to spontaneously perforated pyometra presenting as pneumoperitoneum: report of a case. *Surgery Today*.

[16] Deutchman ME, Hartman KJ (1993). Postpartum pyometra: a case report. *Journal of Family Practice*.

