# Erratum to “Generalized Peritonitis Secondary to Spontaneous Perforation of Pyometra in a 63-Year-Old Patient”

**DOI:** 10.1155/2014/180867

**Published:** 2014-01-09

**Authors:** Ahmed Abu-Zaid, Osama AlOmar, Ahmed Nazer, Ayman Azzam, Zainab Abudan, Ismail A. Al-Badawi

**Affiliations:** ^1^College of Medicine, Alfaisal University, P.O. Box 50927, Riyadh 11533, Saudi Arabia; ^2^Department of Obstetrics and Gynecology, King Faisal Specialist Hospital and Research Center (KFSH&RC), P.O. Box 3354, Riyadh 11211, Saudi Arabia; ^3^Department of General Surgery, Faculty of Medicine, Alexandria University, Alexandria 21526, Egypt

The name of the senior author (Ismail Al-Badawi) was spelled incorrectly and is corrected here as Ismail A. Al-Badawi.

